# *N*,*N*′-Bis(5-bromo-2-hydroxy­benzyl­idene)-2,2-dimethylpropane-1,3-diamine

**DOI:** 10.1107/S160053680802816X

**Published:** 2008-09-06

**Authors:** Hoong-Kun Fun, Reza Kia, Hadi Kargar

**Affiliations:** a X-ray Crystallography Unit, School of Physics, Universiti Sains Malaysia, 11800 USM, Penang, Malaysia; b Department of Chemistry, School of Science, Payame Noor University (PNU), Ardakan, Yazd, Iran

## Abstract

The crystal structure of the title Schiff base compound, C_19_H_20_Br_2_N_2_O_2_, contains two crystallographically independent mol­ecules (*A* and *B*) in the asymmetric unit, with similar conformations. Intra­molecular O—H⋯N (× 4) and C—H⋯N (× 5) hydrogen bonds form six- and five-membered rings, producing *S*(6) and *S*(5) ring motifs, respectively. One of the N atoms in mol­ecule *A* acts as a trifurcated acceptor, the rest of the N atoms being bifurcated acceptors. The dihedral angles between the benzene rings in mol­ecules *A* and *B* are 47.83 (17) and 61.11 (17)°, respectively. The mol­ecular conformation is stabilized by intra­molecular O—H⋯N and C—H⋯N hydrogen bonds. The short distances between the centroids of the benzene rings [3.7799 (19)–3.890 (2) Å] indicate the existence of π–π inter­actions. In addition, the crystal structure is further stabilized by an inter­molecular C—H⋯O hydrogen bond, C—H⋯π inter­actions, and short inter­molecular Br⋯Br and Br⋯O contacts [3.4786 (5) and 3.149 (3) Å, respectively].

## Related literature

For bond-length data, see: Allen *et al.* (1987 ▶). For hydrogen-bond motifs, see: Bernstein *et al.* (1995 ▶). For information on Schiff base ligands and complexes and their applications, see, for example: Fun, Kargar & Kia (2008 ▶); Fun, Kia & Kargar (2008 ▶); Fun, Mirkhani *et al.* (2008*a* ▶,*b* ▶); Calligaris & Randaccio (1987 ▶); Casellato & Vigato (1977 ▶); Pal *et al.* (2005 ▶); Reglinski *et al.* 2004 ▶; Hou *et al.* (2001 ▶); Ren *et al.* (2002 ▶).
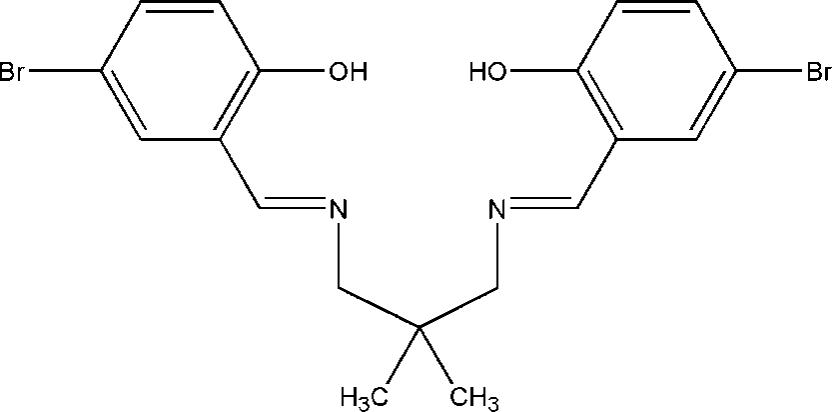


## Experimental

### 

#### Crystal data

C_19_H_20_Br_2_N_2_O_2_
*M* *_r_* = 468.19
Monoclinic, 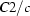

*a* = 31.7684 (10) Å
*b* = 6.2436 (2) Å
*c* = 38.7287 (11) Å
β = 99.870 (2)°
*V* = 7568.1 (4) Å^3^
*Z* = 16
Mo *K*α radiation
μ = 4.30 mm^−1^
*T* = 100.0 (1) K
0.52 × 0.10 × 0.06 mm

#### Data collection

Bruker APEXII CCD area-detector diffractometer
Absorption correction: multi-scan (*SADABS*; Bruker, 2005 ▶) *T*_min_ = 0.213, *T*_max_ = 0.782
47391 measured reflections
11172 independent reflections
6920 reflections with *I* > 2σ(*I*)
*R*_int_ = 0.080

#### Refinement

*R*[*F* ^2^ > 2σ(*F* ^2^)] = 0.043
*wR*(*F* ^2^) = 0.110
*S* = 1.01
11172 reflections
463 parameters
H atoms treated by a mixture of independent and constrained refinement
Δρ_max_ = 0.57 e Å^−3^
Δρ_min_ = −0.46 e Å^−3^

### 

Data collection: *APEX2* (Bruker, 2005 ▶); cell refinement: *SAINT* (Bruker, 2005 ▶); data reduction: *SAINT*; program(s) used to solve structure: *SHELXTL* (Sheldrick, 2008 ▶); program(s) used to refine structure: *SHELXTL*; molecular graphics: *SHELXTL* software used to prepare material for publication: *SHELXTL* and *PLATON* (Spek, 2003 ▶).

## Supplementary Material

Crystal structure: contains datablocks global, I. DOI: 10.1107/S160053680802816X/at2626sup1.cif

Structure factors: contains datablocks I. DOI: 10.1107/S160053680802816X/at2626Isup2.hkl

Additional supplementary materials:  crystallographic information; 3D view; checkCIF report

## Figures and Tables

**Table 1:** Hydrogen-bond geometry (Å, °)

*D*—H⋯*A*	*D*—H	H⋯*A*	*D*⋯*A*	*D*—H⋯*A*
O1*B*—H1*OB*⋯N1*B*	0.85	1.81	2.580 (4)	151
O2*A*—H2*OA*⋯N2*A*	0.85 (4)	1.79 (4)	2.578 (4)	154 (4)
O1*A*—H1*OA*⋯N1*A*	0.79 (5)	1.85 (5)	2.572 (4)	153 (4)
O2*B*—H2*OB*⋯N2*B*	0.73 (5)	1.94 (5)	2.586 (4)	149 (5)
C8*A*—H8*AA*⋯N2*A*	0.99	2.58	2.960 (4)	103
C8*B*—H8*BA*⋯N2*B*	0.99	2.60	2.966 (4)	102
C16*B*—H16*B*⋯O2*B*^i^	0.95	2.58	3.290 (5)	131
C19*A*—H19*B*⋯N1*A*	0.98	2.58	2.918 (4)	100
C19*A*—H19*C*⋯N2*A*	0.98	2.58	2.933 (5)	101
C19*B*—H19*F*⋯N1*B*	0.98	2.60	2.926 (5)	100
C7*B*—H7*BA*⋯*Cg*1^ii^	0.95	2.96	3.571 (4)	123
C18*B*—H18*D*⋯*Cg*2^iii^	0.98	2.77	3.652 (4)	151

Symmetry codes: (i) 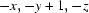; (ii) 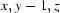; (iii) 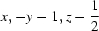. *Cg*1 and *Cg*2 are the centroids of the C1*A*–C6*A* and C12*A*–C17*A* benzene rings, respectively.

**Fig. 1.**
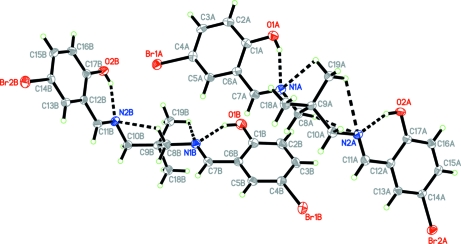
The molecular structure of (I), with atom labels and 50% probability ellipsoids for non-H atoms. Intramolecular interactions are shown as dashed lines.

**Fig. 2.**
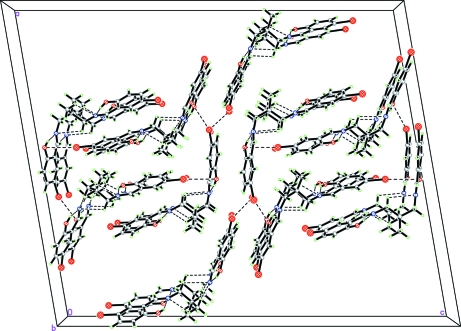
The crystal packing of (I), showing stacking of molecules down the b-axis. Intramolecular and intermolecular interactions are shown as dashed lines.

## supplementary crystallographic information

### Comment

The condensation of primary amines with carbonyl compounds yields Schiff base (Casellato & Vigato, 1977) that are still now regarded as one of the most potential group of chelators for facile preparations of metallo-organic hybrid materials. In the past two decades, the synthesis, structure and properties of Schiff base complexes have stimulated much interest for their noteworthy contributions in single molecule-based magnetism, materials science, catalysis of many reactions like carbonylation, hydroformylation, reduction, oxidation, epoxidation and hydrolysis, *etc.* (Pal *et al.*, 2005; Reglinski *et al.*, 2004; Hou *et al.*, 2001; Ren *et al.*, 2002). Only a relatively small number of free Schiff base ligands have been characterized by X-ray crystallography (Calligaris & Randaccio, 1987). As an extension of our work (Fun, Kargar & Kia, 2008; Fun, Kia & Kargar, 2008; Fun, Mirkhani *et al.*, 2008*a*,*b*) on the structural characterization of Schiff base compounds, the title compound (I), is reported here.

The crystal structure of the title compound (I) (Fig. I), contains two crystallographically independent molecules (A and B) in the asymmetric unit, with similar conformations. The bond lengths and angles are within normal ranges (Allen *et al.*, 1987). Intramolecular O—H···N (*x* 4) and C—H···N (*x* 5) hydrogen bonds form six- and five-membered rings, producing *S*(6) and *S*(5) ring motifs, respectively (Bernstein *et al.* 1995) (Table 1). One of the nitrogen atoms in the molecule A acts as a trifurcated acceptor, but the rest of the nitrogen atoms are bifurcated acceptors. The dihedral angles between the benzene rings in molecule A and B is 47.83 (17)° and 61.11 (17)°. The molecular conformation is stabilized by intramolecular O—H···N and C—H···N hydrogen bonds. The short distances between the centroids of the benzene rings [*Cg*2–*Cg*2 = 3.7799 (19) Å and *Cg*3–*Cg*3 = 3.890 (2) Å] indicate the existence of π–π interactions. The Cg2 and Cg3 are the centroids of the C12A–C17A and C12B–C17B benzene rings. The interesting features of the crystal structure are short intermolecular Br···Br [symmetry code: 1/2 + *x*, -1/2 - *y* + 1/2 + *z*] and Br···O [symmetry code: -*x*, 1 + *y*, 1/2 - *z*] interactions, with distances of 3.4786 (5) and 3.149 (3) Å, respectively, which are significantly shorter than the sum of the van der Waals radii of the relevent atoms.

In addition, the crystal structure is further stabilized by intermolecular C—H···O hydrogen bond and C—H···π interactions.

### Experimental

The synthetic method has been described earlier (Reglinski *et al.*, 2004). Single crystals suitable for X-ray diffraction were obtained by evaporation of an ethanol solution at room temperature.

### Refinement

H atoms bound to the O1A, O2A, and O2B were located in a difference Fourier map and refined freely. H atom bound to O1B was located from a difference Fourier map and constrained to refine with the parent atom after distance restraint of 0.84 (1) Å. The rest of the H atoms were positioned geometrically (C—H = 0.95–0.99 Å) and refined using a riding model.

### Crystal data

**Table:**

C_19_H_20_Br_2_N_2_O_2_	*F*(000) = 3744
*M**_r_* = 468.19	*D*_x_ = 1.644 Mg m^−^^3^
Monoclinic, *C*2/*c*	Mo *K*α radiation, λ = 0.71073 Å
Hall symbol: -C 2yc	Cell parameters from 5640 reflections
*a* = 31.7684 (10) Å	θ = 3.0–27.0°
*b* = 6.2436 (2) Å	µ = 4.30 mm^−^^1^
*c* = 38.7287 (11) Å	*T* = 100 K
β = 99.870 (2)°	Needle, yellow
*V* = 7568.1 (4) Å^3^	0.52 × 0.10 × 0.06 mm
*Z* = 16	

### Data collection

**Table:**

Bruker SMART APEXII CCD area-detector diffractometer	11172 independent reflections
Radiation source: fine-focus sealed tube	6920 reflections with *I* > 2σ(*I*)
graphite	*R*_int_ = 0.080
φ and ω scans	θ_max_ = 30.2°, θ_min_ = 1.1°
Absorption correction: multi-scan (SADABS; Bruker, 2005)	*h* = −36→44
*T*_min_ = 0.213, *T*_max_ = 0.783	*k* = −8→8
47391 measured reflections	*l* = −54→54

### Refinement

**Table:**

Refinement on *F*^2^	Primary atom site location: structure-invariant direct methods
Least-squares matrix: full	Secondary atom site location: difference Fourier map
*R*[*F*^2^ > 2σ(*F*^2^)] = 0.043	Hydrogen site location: inferred from neighbouring sites
*wR*(*F*^2^) = 0.110	H atoms treated by a mixture of independent and constrained refinement
*S* = 1.01	*w* = 1/[σ^2^(*F*_o_^2^) + (0.0443*P*)^2^ + 4.1476*P*] where *P* = (*F*_o_^2^ + 2*F*_c_^2^)/3
11172 reflections	(Δ/σ)_max_ = 0.001
463 parameters	Δρ_max_ = 0.57 e Å^−^^3^
0 restraints	Δρ_min_ = −0.46 e Å^−^^3^

### Special details

**Table:**

Experimental. The low-temperature data was collected with the Oxford Cyrosystem Cobra low-temperature attachment.
Geometry. All esds (except the esd in the dihedral angle between two l.s. planes) are estimated using the full covariance matrix. The cell esds are taken into account individually in the estimation of esds in distances, angles and torsion angles; correlations between esds in cell parameters are only used when they are defined by crystal symmetry. An approximate (isotropic) treatment of cell esds is used for estimating esds involving l.s. planes.
Refinement. Refinement of F^2^ against ALL reflections. The weighted R-factor wR and goodness of fit S are based on F^2^, conventional R-factors R are based on F, with F set to zero for negative F^2^. The threshold expression of F^2^ > 2sigma(F^2^) is used only for calculating R-factors(gt) etc. and is not relevant to the choice of reflections for refinement. R-factors based on F^2^ are statistically about twice as large as those based on F, and R- factors based on ALL data will be even larger.

### Fractional atomic coordinates and isotropic or equivalent isotropic displacement parameters (Å^2^)

**Table:**

	*x*	*y*	*z*	*U*_iso_*/*U*_eq_	
Br1A	0.048763 (12)	0.47249 (7)	0.124135 (9)	0.02437 (10)	
Br2A	0.330044 (11)	0.00679 (6)	0.479516 (9)	0.02179 (9)	
O1A	0.06595 (9)	0.8408 (5)	0.26946 (7)	0.0269 (6)	
O2A	0.18364 (8)	0.6081 (4)	0.43058 (7)	0.0214 (6)	
N1A	0.09915 (9)	0.4834 (5)	0.29342 (7)	0.0207 (7)	
N2A	0.14171 (9)	0.2889 (5)	0.39950 (7)	0.0185 (6)	
C1A	0.06195 (11)	0.7509 (6)	0.23744 (9)	0.0197 (8)	
C2A	0.04274 (11)	0.8701 (6)	0.20872 (10)	0.0231 (8)	
H2AA	0.0321	1.0093	0.2121	0.028*	
C3A	0.03900 (11)	0.7879 (6)	0.17532 (9)	0.0206 (8)	
H3AA	0.0261	0.8709	0.1558	0.025*	
C4A	0.05410 (11)	0.5838 (6)	0.17031 (9)	0.0202 (8)	
C5A	0.07244 (11)	0.4615 (6)	0.19833 (9)	0.0191 (8)	
H5AA	0.0825	0.3215	0.1946	0.023*	
C6A	0.07639 (11)	0.5435 (6)	0.23258 (9)	0.0182 (8)	
C7A	0.09411 (11)	0.4094 (6)	0.26223 (9)	0.0197 (8)	
H7AA	0.1020	0.2657	0.2584	0.024*	
C8A	0.11469 (11)	0.3431 (6)	0.32298 (9)	0.0200 (8)	
H8AA	0.1426	0.3959	0.3353	0.024*	
H8AB	0.1189	0.1969	0.3143	0.024*	
C9A	0.08291 (11)	0.3352 (6)	0.34871 (9)	0.0178 (7)	
C10A	0.10260 (11)	0.1953 (6)	0.37987 (9)	0.0191 (8)	
H10A	0.0816	0.1765	0.3958	0.023*	
H10B	0.1091	0.0520	0.3712	0.023*	
C11A	0.17384 (11)	0.1677 (6)	0.40944 (8)	0.0182 (8)	
H11A	0.1724	0.0207	0.4030	0.022*	
C12A	0.21289 (11)	0.2529 (6)	0.43068 (8)	0.0168 (7)	
C13A	0.24770 (11)	0.1167 (6)	0.44173 (8)	0.0174 (7)	
H13A	0.2467	−0.0279	0.4340	0.021*	
C14A	0.28333 (10)	0.1924 (6)	0.46376 (9)	0.0166 (7)	
C15A	0.28534 (11)	0.4037 (6)	0.47528 (8)	0.0187 (8)	
H15A	0.3097	0.4540	0.4909	0.022*	
C16A	0.25179 (11)	0.5388 (6)	0.46392 (9)	0.0191 (8)	
H16A	0.2534	0.6836	0.4715	0.023*	
C17A	0.21539 (11)	0.4681 (6)	0.44144 (9)	0.0170 (7)	
C18A	0.04145 (11)	0.2283 (7)	0.33119 (10)	0.0235 (8)	
H18A	0.0211	0.2254	0.3476	0.035*	
H18B	0.0475	0.0814	0.3245	0.035*	
H18C	0.0291	0.3094	0.3102	0.035*	
C19A	0.07370 (12)	0.5588 (6)	0.36131 (9)	0.0222 (8)	
H19A	0.0532	0.5491	0.3775	0.033*	
H19B	0.0617	0.6476	0.3412	0.033*	
H19C	0.1003	0.6235	0.3734	0.033*	
Br1B	0.203156 (14)	−0.21813 (7)	0.339930 (10)	0.03229 (11)	
Br2B	−0.107371 (12)	−0.17661 (7)	0.040046 (10)	0.02582 (10)	
O1B	0.18949 (9)	0.3706 (4)	0.21548 (7)	0.0311 (7)	
H1OB	0.1733	0.3089	0.1988	0.047*	
O2B	0.04871 (9)	0.3734 (5)	0.04206 (7)	0.0234 (6)	
N1B	0.15040 (9)	0.0685 (5)	0.17729 (7)	0.0210 (7)	
N2B	0.09435 (9)	0.0503 (5)	0.06810 (7)	0.0203 (7)	
C1B	0.19306 (11)	0.2322 (6)	0.24240 (10)	0.0230 (8)	
C2B	0.21359 (12)	0.2995 (7)	0.27530 (10)	0.0274 (9)	
H2BA	0.2255	0.4392	0.2781	0.033*	
C3B	0.21669 (12)	0.1653 (7)	0.30373 (10)	0.0260 (9)	
H3BA	0.2306	0.2126	0.3261	0.031*	
C4B	0.19961 (12)	−0.0384 (7)	0.29977 (9)	0.0241 (9)	
C5B	0.17993 (11)	−0.1126 (6)	0.26741 (9)	0.0205 (8)	
H5BA	0.1687	−0.2539	0.2650	0.025*	
C6B	0.17662 (11)	0.0223 (6)	0.23812 (9)	0.0195 (8)	
C7B	0.15596 (11)	−0.0552 (6)	0.20381 (9)	0.0185 (8)	
H7BA	0.1466	−0.1997	0.2012	0.022*	
C8B	0.13053 (11)	−0.0151 (6)	0.14318 (9)	0.0220 (8)	
H8BA	0.1022	0.0522	0.1360	0.026*	
H8BB	0.1263	−0.1716	0.1449	0.026*	
C9B	0.15859 (11)	0.0308 (6)	0.11529 (9)	0.0202 (8)	
C10B	0.13537 (11)	−0.0555 (7)	0.08002 (9)	0.0218 (8)	
H10C	0.1539	−0.0355	0.0621	0.026*	
H10D	0.1305	−0.2111	0.0822	0.026*	
C11B	0.06085 (11)	−0.0637 (6)	0.06348 (9)	0.0193 (8)	
H11B	0.0632	−0.2135	0.0676	0.023*	
C12B	0.01870 (11)	0.0298 (6)	0.05198 (8)	0.0175 (7)	
C13B	−0.01786 (11)	−0.0943 (6)	0.05134 (9)	0.0203 (8)	
H13B	−0.0154	−0.2390	0.0590	0.024*	
C14B	−0.05760 (11)	−0.0080 (6)	0.03961 (9)	0.0188 (8)	
C15B	−0.06189 (11)	0.2017 (6)	0.02785 (9)	0.0213 (8)	
H15B	−0.0894	0.2590	0.0193	0.026*	
C16B	−0.02609 (11)	0.3268 (6)	0.02854 (9)	0.0212 (8)	
H16B	−0.0290	0.4705	0.0204	0.025*	
C17B	0.01438 (11)	0.2447 (6)	0.04102 (9)	0.0192 (8)	
C18B	0.20099 (11)	−0.0906 (7)	0.12457 (10)	0.0249 (9)	
H18D	0.2190	−0.0598	0.1070	0.037*	
H18E	0.1953	−0.2448	0.1250	0.037*	
H18F	0.2158	−0.0447	0.1477	0.037*	
C19B	0.16738 (12)	0.2702 (6)	0.11289 (10)	0.0239 (8)	
H19D	0.1402	0.3474	0.1069	0.036*	
H19E	0.1849	0.2955	0.0948	0.036*	
H19F	0.1827	0.3215	0.1355	0.036*	
H2OA	0.1637 (13)	0.531 (7)	0.4195 (10)	0.027 (12)*	
H1OA	0.0731 (13)	0.745 (8)	0.2823 (11)	0.031 (14)*	
H2OB	0.0676 (14)	0.317 (8)	0.0506 (12)	0.043 (16)*	

### Atomic displacement parameters (Å^2^)

**Table:**

	*U*^11^	*U*^22^	*U*^33^	*U*^12^	*U*^13^	*U*^23^
Br1A	0.02596 (19)	0.0293 (2)	0.01827 (17)	0.00233 (18)	0.00511 (14)	0.00038 (16)
Br2A	0.01765 (17)	0.0224 (2)	0.02391 (17)	0.00251 (16)	−0.00050 (13)	0.00215 (16)
O1A	0.0358 (16)	0.0207 (16)	0.0234 (14)	0.0043 (14)	0.0028 (12)	−0.0006 (13)
O2A	0.0205 (13)	0.0148 (14)	0.0271 (13)	0.0027 (12)	−0.0014 (11)	−0.0022 (12)
N1A	0.0187 (15)	0.0206 (17)	0.0223 (14)	0.0010 (14)	0.0022 (12)	0.0022 (14)
N2A	0.0204 (15)	0.0166 (17)	0.0176 (14)	0.0015 (14)	0.0008 (12)	0.0022 (13)
C1A	0.0174 (17)	0.019 (2)	0.0241 (18)	−0.0018 (16)	0.0064 (14)	−0.0020 (16)
C2A	0.0231 (19)	0.017 (2)	0.030 (2)	0.0026 (17)	0.0064 (16)	0.0017 (17)
C3A	0.0163 (17)	0.021 (2)	0.0241 (18)	0.0019 (16)	0.0028 (14)	0.0081 (16)
C4A	0.0179 (17)	0.026 (2)	0.0179 (16)	−0.0035 (17)	0.0051 (14)	−0.0008 (16)
C5A	0.0158 (16)	0.017 (2)	0.0247 (18)	0.0005 (15)	0.0050 (14)	−0.0001 (16)
C6A	0.0155 (17)	0.0166 (19)	0.0224 (17)	−0.0002 (15)	0.0033 (13)	0.0023 (15)
C7A	0.0177 (18)	0.019 (2)	0.0235 (18)	0.0010 (16)	0.0055 (14)	−0.0019 (16)
C8A	0.0171 (17)	0.022 (2)	0.0195 (17)	−0.0012 (16)	0.0009 (14)	−0.0003 (16)
C9A	0.0184 (17)	0.0150 (19)	0.0192 (16)	0.0017 (16)	0.0008 (13)	0.0016 (15)
C10A	0.0166 (17)	0.018 (2)	0.0226 (17)	−0.0039 (16)	0.0030 (14)	−0.0010 (16)
C11A	0.0228 (18)	0.0174 (19)	0.0153 (16)	−0.0026 (16)	0.0057 (14)	0.0023 (15)
C12A	0.0161 (17)	0.0189 (19)	0.0156 (15)	−0.0010 (15)	0.0038 (13)	0.0017 (15)
C13A	0.0210 (18)	0.0150 (18)	0.0168 (16)	0.0014 (16)	0.0046 (13)	0.0014 (15)
C14A	0.0156 (16)	0.0180 (19)	0.0165 (15)	0.0023 (15)	0.0035 (13)	0.0048 (15)
C15A	0.0163 (17)	0.024 (2)	0.0154 (16)	−0.0002 (16)	0.0011 (13)	0.0013 (15)
C16A	0.0221 (18)	0.0167 (19)	0.0189 (16)	−0.0033 (16)	0.0044 (14)	−0.0019 (15)
C17A	0.0180 (17)	0.0147 (19)	0.0189 (16)	0.0001 (15)	0.0053 (13)	−0.0003 (15)
C18A	0.0173 (18)	0.024 (2)	0.0273 (19)	−0.0035 (17)	−0.0007 (15)	−0.0016 (17)
C19A	0.0231 (19)	0.020 (2)	0.0219 (17)	0.0019 (17)	−0.0021 (14)	−0.0003 (16)
Br1B	0.0397 (2)	0.0322 (3)	0.02182 (19)	0.0007 (2)	−0.00362 (16)	−0.00159 (18)
Br2B	0.01964 (18)	0.0264 (2)	0.0304 (2)	−0.00490 (17)	0.00142 (15)	−0.00229 (17)
O1B	0.0361 (16)	0.0214 (15)	0.0344 (15)	−0.0074 (13)	0.0022 (13)	0.0013 (13)
O2B	0.0202 (14)	0.0190 (16)	0.0299 (14)	−0.0024 (13)	0.0015 (12)	0.0058 (12)
N1B	0.0205 (16)	0.0222 (18)	0.0203 (15)	0.0028 (14)	0.0035 (12)	0.0007 (14)
N2B	0.0181 (15)	0.0252 (18)	0.0178 (14)	0.0016 (14)	0.0035 (11)	0.0028 (13)
C1B	0.0184 (18)	0.021 (2)	0.0294 (19)	−0.0007 (17)	0.0048 (15)	−0.0011 (17)
C2B	0.022 (2)	0.020 (2)	0.038 (2)	−0.0057 (18)	0.0029 (17)	−0.0074 (19)
C3B	0.0218 (19)	0.025 (2)	0.029 (2)	−0.0002 (18)	−0.0016 (15)	−0.0111 (18)
C4B	0.0221 (19)	0.027 (2)	0.0227 (17)	0.0073 (17)	0.0024 (15)	−0.0021 (17)
C5B	0.0186 (18)	0.0170 (19)	0.0257 (18)	0.0010 (16)	0.0036 (14)	−0.0027 (16)
C6B	0.0169 (17)	0.019 (2)	0.0221 (17)	0.0030 (16)	0.0030 (13)	−0.0014 (16)
C7B	0.0150 (17)	0.0178 (19)	0.0234 (17)	−0.0018 (15)	0.0052 (14)	−0.0034 (16)
C8B	0.0181 (17)	0.023 (2)	0.0243 (17)	−0.0025 (17)	0.0023 (14)	0.0014 (17)
C9B	0.0155 (17)	0.022 (2)	0.0234 (17)	0.0019 (16)	0.0030 (14)	0.0031 (16)
C10B	0.0210 (18)	0.024 (2)	0.0204 (17)	0.0026 (17)	0.0037 (14)	0.0003 (16)
C11B	0.0200 (18)	0.019 (2)	0.0194 (17)	0.0048 (16)	0.0037 (14)	0.0011 (15)
C12B	0.0171 (17)	0.020 (2)	0.0149 (15)	0.0001 (16)	0.0004 (13)	−0.0013 (15)
C13B	0.0222 (19)	0.018 (2)	0.0203 (17)	0.0013 (16)	0.0020 (14)	0.0023 (16)
C14B	0.0170 (17)	0.020 (2)	0.0187 (16)	−0.0024 (16)	0.0025 (13)	−0.0053 (16)
C15B	0.0184 (18)	0.024 (2)	0.0207 (17)	0.0037 (17)	−0.0004 (14)	0.0014 (16)
C16B	0.026 (2)	0.017 (2)	0.0205 (17)	0.0018 (17)	0.0038 (15)	0.0012 (16)
C17B	0.0231 (19)	0.019 (2)	0.0162 (16)	−0.0003 (16)	0.0048 (14)	−0.0010 (15)
C18B	0.0200 (19)	0.028 (2)	0.0261 (19)	0.0019 (18)	0.0016 (15)	−0.0024 (18)
C19B	0.0210 (19)	0.024 (2)	0.0267 (19)	−0.0024 (17)	0.0050 (15)	0.0042 (17)

### Geometric parameters (Å, °)

**Table:**

Br1A—C4A	1.899 (3)	Br1B—C4B	1.906 (4)
Br2A—C14A	1.899 (3)	Br2B—C14B	1.902 (4)
O1A—C1A	1.347 (4)	O1B—C1B	1.344 (5)
O1A—H1OA	0.79 (4)	O1B—H1OB	0.8464
O2A—C17A	1.347 (4)	O2B—C17B	1.350 (4)
O2A—H2OA	0.85 (4)	O2B—H2OB	0.73 (4)
N1A—C7A	1.278 (4)	N1B—C7B	1.273 (4)
N1A—C8A	1.459 (4)	N1B—C8B	1.460 (4)
N2A—C11A	1.276 (4)	N2B—C11B	1.267 (5)
N2A—C10A	1.462 (4)	N2B—C10B	1.463 (5)
C1A—C2A	1.390 (5)	C1B—C2B	1.393 (5)
C1A—C6A	1.397 (5)	C1B—C6B	1.410 (5)
C2A—C3A	1.378 (5)	C2B—C3B	1.374 (6)
C2A—H2AA	0.9500	C2B—H2BA	0.9500
C3A—C4A	1.387 (5)	C3B—C4B	1.381 (6)
C3A—H3AA	0.9500	C3B—H3BA	0.9500
C4A—C5A	1.372 (5)	C4B—C5B	1.381 (5)
C5A—C6A	1.407 (5)	C5B—C6B	1.402 (5)
C5A—H5AA	0.9500	C5B—H5BA	0.9500
C6A—C7A	1.454 (5)	C6B—C7B	1.460 (5)
C7A—H7AA	0.9500	C7B—H7BA	0.9500
C8A—C9A	1.536 (5)	C8B—C9B	1.540 (5)
C8A—H8AA	0.9900	C8B—H8BA	0.9900
C8A—H8AB	0.9900	C8B—H8BB	0.9900
C9A—C19A	1.524 (5)	C9B—C19B	1.527 (5)
C9A—C18A	1.528 (5)	C9B—C18B	1.533 (5)
C9A—C10A	1.534 (5)	C9B—C10B	1.534 (5)
C10A—H10A	0.9900	C10B—H10C	0.9900
C10A—H10B	0.9900	C10B—H10D	0.9900
C11A—C12A	1.466 (5)	C11B—C12B	1.458 (5)
C11A—H11A	0.9500	C11B—H11B	0.9500
C12A—C13A	1.403 (5)	C12B—C13B	1.393 (5)
C12A—C17A	1.405 (5)	C12B—C17B	1.407 (5)
C13A—C14A	1.379 (5)	C13B—C14B	1.376 (5)
C13A—H13A	0.9500	C13B—H13B	0.9500
C14A—C15A	1.391 (5)	C14B—C15B	1.385 (5)
C15A—C16A	1.371 (5)	C15B—C16B	1.376 (5)
C15A—H15A	0.9500	C15B—H15B	0.9500
C16A—C17A	1.395 (5)	C16B—C17B	1.391 (5)
C16A—H16A	0.9500	C16B—H16B	0.9500
C18A—H18A	0.9800	C18B—H18D	0.9800
C18A—H18B	0.9800	C18B—H18E	0.9800
C18A—H18C	0.9800	C18B—H18F	0.9800
C19A—H19A	0.9800	C19B—H19D	0.9800
C19A—H19B	0.9800	C19B—H19E	0.9800
C19A—H19C	0.9800	C19B—H19F	0.9800
			
C1A—O1A—H1OA	104 (3)	C1B—O1B—H1OB	105.1
C17A—O2A—H2OA	104 (3)	C17B—O2B—H2OB	109 (4)
C7A—N1A—C8A	119.7 (3)	C7B—N1B—C8B	119.5 (3)
C11A—N2A—C10A	119.0 (3)	C11B—N2B—C10B	118.2 (3)
O1A—C1A—C2A	118.2 (3)	O1B—C1B—C2B	118.7 (4)
O1A—C1A—C6A	121.9 (3)	O1B—C1B—C6B	121.7 (3)
C2A—C1A—C6A	119.9 (3)	C2B—C1B—C6B	119.6 (4)
C3A—C2A—C1A	120.5 (4)	C3B—C2B—C1B	120.5 (4)
C3A—C2A—H2AA	119.7	C3B—C2B—H2BA	119.7
C1A—C2A—H2AA	119.7	C1B—C2B—H2BA	119.8
C2A—C3A—C4A	119.8 (3)	C2B—C3B—C4B	120.0 (3)
C2A—C3A—H3AA	120.1	C2B—C3B—H3BA	120.0
C4A—C3A—H3AA	120.1	C4B—C3B—H3BA	120.0
C5A—C4A—C3A	120.7 (3)	C3B—C4B—C5B	121.2 (4)
C5A—C4A—Br1A	119.9 (3)	C3B—C4B—Br1B	119.0 (3)
C3A—C4A—Br1A	119.4 (3)	C5B—C4B—Br1B	119.8 (3)
C4A—C5A—C6A	120.0 (3)	C4B—C5B—C6B	119.5 (4)
C4A—C5A—H5AA	120.0	C4B—C5B—H5BA	120.2
C6A—C5A—H5AA	120.0	C6B—C5B—H5BA	120.2
C1A—C6A—C5A	119.1 (3)	C5B—C6B—C1B	119.2 (3)
C1A—C6A—C7A	121.3 (3)	C5B—C6B—C7B	119.8 (3)
C5A—C6A—C7A	119.7 (3)	C1B—C6B—C7B	121.0 (3)
N1A—C7A—C6A	120.5 (4)	N1B—C7B—C6B	120.8 (3)
N1A—C7A—H7AA	119.8	N1B—C7B—H7BA	119.6
C6A—C7A—H7AA	119.8	C6B—C7B—H7BA	119.6
N1A—C8A—C9A	110.9 (3)	N1B—C8B—C9B	110.8 (3)
N1A—C8A—H8AA	109.5	N1B—C8B—H8BA	109.5
C9A—C8A—H8AA	109.5	C9B—C8B—H8BA	109.5
N1A—C8A—H8AB	109.5	N1B—C8B—H8BB	109.5
C9A—C8A—H8AB	109.5	C9B—C8B—H8BB	109.5
H8AA—C8A—H8AB	108.0	H8BA—C8B—H8BB	108.1
C19A—C9A—C18A	110.1 (3)	C19B—C9B—C18B	109.6 (3)
C19A—C9A—C10A	110.2 (3)	C19B—C9B—C10B	110.8 (3)
C18A—C9A—C10A	107.8 (3)	C18B—C9B—C10B	107.7 (3)
C19A—C9A—C8A	111.2 (3)	C19B—C9B—C8B	111.0 (3)
C18A—C9A—C8A	109.8 (3)	C18B—C9B—C8B	109.5 (3)
C10A—C9A—C8A	107.7 (3)	C10B—C9B—C8B	108.1 (3)
N2A—C10A—C9A	112.1 (3)	N2B—C10B—C9B	112.9 (3)
N2A—C10A—H10A	109.2	N2B—C10B—H10C	109.0
C9A—C10A—H10A	109.2	C9B—C10B—H10C	109.0
N2A—C10A—H10B	109.2	N2B—C10B—H10D	109.0
C9A—C10A—H10B	109.2	C9B—C10B—H10D	109.0
H10A—C10A—H10B	107.9	H10C—C10B—H10D	107.8
N2A—C11A—C12A	120.7 (3)	N2B—C11B—C12B	121.6 (4)
N2A—C11A—H11A	119.7	N2B—C11B—H11B	119.2
C12A—C11A—H11A	119.7	C12B—C11B—H11B	119.2
C13A—C12A—C17A	119.2 (3)	C13B—C12B—C17B	119.1 (3)
C13A—C12A—C11A	119.9 (3)	C13B—C12B—C11B	120.1 (3)
C17A—C12A—C11A	120.9 (3)	C17B—C12B—C11B	120.7 (3)
C14A—C13A—C12A	120.1 (3)	C14B—C13B—C12B	120.2 (4)
C14A—C13A—H13A	119.9	C14B—C13B—H13B	119.9
C12A—C13A—H13A	119.9	C12B—C13B—H13B	119.9
C13A—C14A—C15A	120.8 (3)	C13B—C14B—C15B	120.8 (3)
C13A—C14A—Br2A	120.2 (3)	C13B—C14B—Br2B	119.8 (3)
C15A—C14A—Br2A	119.0 (2)	C15B—C14B—Br2B	119.4 (3)
C16A—C15A—C14A	119.3 (3)	C16B—C15B—C14B	119.7 (3)
C16A—C15A—H15A	120.3	C16B—C15B—H15B	120.1
C14A—C15A—H15A	120.3	C14B—C15B—H15B	120.1
C15A—C16A—C17A	121.4 (3)	C15B—C16B—C17B	120.6 (4)
C15A—C16A—H16A	119.3	C15B—C16B—H16B	119.7
C17A—C16A—H16A	119.3	C17B—C16B—H16B	119.7
O2A—C17A—C16A	119.1 (3)	O2B—C17B—C16B	119.0 (3)
O2A—C17A—C12A	121.8 (3)	O2B—C17B—C12B	121.5 (3)
C16A—C17A—C12A	119.1 (3)	C16B—C17B—C12B	119.5 (3)
C9A—C18A—H18A	109.5	C9B—C18B—H18D	109.5
C9A—C18A—H18B	109.5	C9B—C18B—H18E	109.5
H18A—C18A—H18B	109.5	H18D—C18B—H18E	109.5
C9A—C18A—H18C	109.5	C9B—C18B—H18F	109.5
H18A—C18A—H18C	109.5	H18D—C18B—H18F	109.5
H18B—C18A—H18C	109.5	H18E—C18B—H18F	109.5
C9A—C19A—H19A	109.5	C9B—C19B—H19D	109.5
C9A—C19A—H19B	109.5	C9B—C19B—H19E	109.5
H19A—C19A—H19B	109.5	H19D—C19B—H19E	109.5
C9A—C19A—H19C	109.5	C9B—C19B—H19F	109.5
H19A—C19A—H19C	109.5	H19D—C19B—H19F	109.5
H19B—C19A—H19C	109.5	H19E—C19B—H19F	109.5
			
O1A—C1A—C2A—C3A	178.1 (3)	O1B—C1B—C2B—C3B	177.9 (3)
C6A—C1A—C2A—C3A	−2.1 (5)	C6B—C1B—C2B—C3B	−2.1 (6)
C1A—C2A—C3A—C4A	0.7 (6)	C1B—C2B—C3B—C4B	0.4 (6)
C2A—C3A—C4A—C5A	0.6 (5)	C2B—C3B—C4B—C5B	1.2 (6)
C2A—C3A—C4A—Br1A	−180.0 (3)	C2B—C3B—C4B—Br1B	−178.4 (3)
C3A—C4A—C5A—C6A	−0.5 (5)	C3B—C4B—C5B—C6B	−1.0 (6)
Br1A—C4A—C5A—C6A	−179.9 (3)	Br1B—C4B—C5B—C6B	178.6 (3)
O1A—C1A—C6A—C5A	−178.0 (3)	C4B—C5B—C6B—C1B	−0.7 (5)
C2A—C1A—C6A—C5A	2.1 (5)	C4B—C5B—C6B—C7B	179.8 (3)
O1A—C1A—C6A—C7A	3.9 (5)	O1B—C1B—C6B—C5B	−177.7 (3)
C2A—C1A—C6A—C7A	−176.0 (3)	C2B—C1B—C6B—C5B	2.2 (5)
C4A—C5A—C6A—C1A	−0.9 (5)	O1B—C1B—C6B—C7B	1.7 (5)
C4A—C5A—C6A—C7A	177.2 (3)	C2B—C1B—C6B—C7B	−178.3 (3)
C8A—N1A—C7A—C6A	176.5 (3)	C8B—N1B—C7B—C6B	178.6 (3)
C1A—C6A—C7A—N1A	−4.1 (5)	C5B—C6B—C7B—N1B	175.9 (3)
C5A—C6A—C7A—N1A	177.8 (3)	C1B—C6B—C7B—N1B	−3.6 (5)
C7A—N1A—C8A—C9A	−122.4 (4)	C7B—N1B—C8B—C9B	−126.7 (4)
N1A—C8A—C9A—C19A	−56.1 (4)	N1B—C8B—C9B—C19B	−56.8 (4)
N1A—C8A—C9A—C18A	66.0 (4)	N1B—C8B—C9B—C18B	64.4 (4)
N1A—C8A—C9A—C10A	−176.9 (3)	N1B—C8B—C9B—C10B	−178.5 (3)
C11A—N2A—C10A—C9A	−136.7 (3)	C11B—N2B—C10B—C9B	−119.8 (4)
C19A—C9A—C10A—N2A	−57.3 (4)	C19B—C9B—C10B—N2B	−59.5 (4)
C18A—C9A—C10A—N2A	−177.5 (3)	C18B—C9B—C10B—N2B	−179.4 (3)
C8A—C9A—C10A—N2A	64.2 (4)	C8B—C9B—C10B—N2B	62.3 (4)
C10A—N2A—C11A—C12A	−176.9 (3)	C10B—N2B—C11B—C12B	179.4 (3)
N2A—C11A—C12A—C13A	179.2 (3)	N2B—C11B—C12B—C13B	−172.0 (3)
N2A—C11A—C12A—C17A	1.9 (5)	N2B—C11B—C12B—C17B	8.8 (5)
C17A—C12A—C13A—C14A	2.0 (5)	C17B—C12B—C13B—C14B	1.0 (5)
C11A—C12A—C13A—C14A	−175.4 (3)	C11B—C12B—C13B—C14B	−178.2 (3)
C12A—C13A—C14A—C15A	−0.1 (5)	C12B—C13B—C14B—C15B	0.9 (5)
C12A—C13A—C14A—Br2A	178.7 (3)	C12B—C13B—C14B—Br2B	−178.3 (3)
C13A—C14A—C15A—C16A	−1.4 (5)	C13B—C14B—C15B—C16B	−1.3 (5)
Br2A—C14A—C15A—C16A	179.8 (3)	Br2B—C14B—C15B—C16B	177.9 (3)
C14A—C15A—C16A—C17A	0.9 (5)	C14B—C15B—C16B—C17B	−0.2 (5)
C15A—C16A—C17A—O2A	−178.9 (3)	C15B—C16B—C17B—O2B	−178.5 (3)
C15A—C16A—C17A—C12A	1.0 (5)	C15B—C16B—C17B—C12B	2.0 (5)
C13A—C12A—C17A—O2A	177.5 (3)	C13B—C12B—C17B—O2B	178.1 (3)
C11A—C12A—C17A—O2A	−5.2 (5)	C11B—C12B—C17B—O2B	−2.7 (5)
C13A—C12A—C17A—C16A	−2.4 (5)	C13B—C12B—C17B—C16B	−2.4 (5)
C11A—C12A—C17A—C16A	174.9 (3)	C11B—C12B—C17B—C16B	176.8 (3)

### Hydrogen-bond geometry (Å, °)

**Table:**

*D*—H···*A*	*D*—H	H···*A*	*D*···*A*	*D*—H···*A*
O1B—H1OB···N1B	0.85	1.81	2.580 (4)	151.
O2A—H2OA···N2A	0.85 (4)	1.79 (4)	2.578 (4)	154 (4)
O1A—H1OA···N1A	0.79 (5)	1.85 (5)	2.572 (4)	153 (4)
O2B—H2OB···N2B	0.73 (5)	1.94 (5)	2.586 (4)	149 (5)
C8A—H8AA···N2A	0.99	2.58	2.960 (4)	103.
C8B—H8BA···N2B	0.99	2.60	2.966 (4)	102.
C16B—H16B···O2B^i^	0.95	2.58	3.290 (5)	131.
C19A—H19B···N1A	0.98	2.58	2.918 (4)	100.
C19A—H19C···N2A	0.98	2.58	2.933 (5)	101.
C19B—H19F···N1B	0.98	2.60	2.926 (5)	100.
C7B—H7BA···Cg1^ii^	0.95	2.96	3.571 (4)	123.
C18B—H18D···Cg2^iii^	0.98	2.77	3.652 (4)	151.

Symmetry codes: (i) −*x*, −*y*+1, −*z*; (ii) *x*, *y*−1, *z*; (iii) *x*, −*y*−1, *z*−1/2.
